# Association of PD-L1 expression and clinical outcomes in ROS1 - rearranged advanced non-small cell lung cancer treated with crizotinib

**DOI:** 10.3389/fonc.2024.1405683

**Published:** 2024-05-21

**Authors:** Huixian Zhang, Ziheng Zhang, Ningning Yan, Xingya Li

**Affiliations:** ^1^ Department of Medical Oncology, The First Affiliated Hospital of Zhengzhou University, Zhengzhou, Henan, China; ^2^ Department of Emergency Medicine, The Fifth Affiliated Hospital of Zhengzhou University, Zhengzhou, Henan, China

**Keywords:** PD-L1, clinical outcomes, ROS1, NSCLC, crizotinib

## Abstract

**Background:**

Programmed cell death ligand 1 (PD-L1) is more readily expressed in ROS proto-oncogene 1 (ROS1) rearranged non-small cell lung cancer (NSCLC) compared to NSCLC cases lacking driver gene mutations. Prior research has established a link between PD-L1 expression and reduced effectiveness of EGFR or ALK inhibitors in EGFR or ALK-positive NSCLC. Nonetheless, the relationship between initial PD-L1 levels and the clinical impact of first-line crizotinib therapy in ROS1-rearranged NSCLC is still uncertain.

**Methods:**

From January 2016 to December 2021, a total of 246 patients with ROS1 positive tumors were collected. Out of these, 82 patients with advanced ROS1-rearranged NSCLC, who were treated with crizotinib as their initial therapy, were selected for the study. The study aimed primarily to evaluate the objective response rate (ORR) and progression-free survival (PFS), and secondarily to assess disease control rate (DCR) and overall survival (OS).

**Results:**

Of the 82 advanced ROS1-rearranged NSCLC patients, 38 exhibited PD-L1 positivity, subdivided into 11 with high and 27 with low expression levels, while the remaining 44 showed no PD-L1 expression. The ORR for all included patients was 80.5%. No statistically significant variance in ORR was observed among ROS1-rearranged NSCLC patients across differing PD-L1 expression statuses. However, there was a statistically significant difference in DCR between PD-L1 negative group (100%) and high expression group (90.9%) (p=0.04). The median PFS spanned 26.4 months for the PD-L1 negative group, 16.6 for the low expression group, and 13.7 for the high expression group (p=0.001). Additionally, a notable statistical disparity was also observed in median PFS between the PD-L1 negative and positive groups (p=0.02). For the entire study population, the median OS was 53.0 months (95% CI 43.8 - 62.2). In the PD-L1-negative group, the median OS reached 57.2 months, compared to 53.0 months in the PD-L1-positive group, a difference lacking statistical significance (p=0.43).

**Conclusions:**

Our results suggest that for ROS1-positive NSCLC patients receiving crizotinib as first-line therapy, PD-L1 expression may serve as a negative prognostic marker for PFS rather than OS.

## 1 Introduction

Lung cancer is currently the most common cause of cancer death worldwide (1). Non-small cell lung cancer (NSCLC) accounts for 85% of all lung cancer cases, of which more than 50% of lung cancer at the time of diagnosis has been distant metastasis, and only 20% to 25% of NSCLC cancers have the opportunity for surgery (2). The evolution of molecular targeted therapy and immunotherapy has notably prolonged the overall survival in patients with advanced NSCLC (3, 4).

The ROS proto-oncogene 1 (ROS1) is encoded by the ROS1 gene, situated on chromosome 6q22.1, and belongs to the tyrosine kinase insulin receptor subfamily (5). ROS1 rearrangement have subsequently been identified in a variety of tumor types, including 1%-2% of NSCLCs (6, 7). Such rearrangements result in the fusion of a segment of ROS1, encompassing the entire tyrosine kinase domain, with one of several different partner proteins (8). Notably, tumors with ROS1 rearrangements and those with anaplastic lymphoma kinase (ALK) rearrangements in NSCLC share similar clinical characteristics (9). ROS1 rearrangements are more prevalent among light smokers or non-smokers (10). Although lung adenocarcinoma represents the most common histological type, ROS1 fusion has also been reported in large cell and squamous tissues (11). Crizotinib, endorsed by the U.S. Food and Drug Administration (FDA) in 2016, was the inaugural tyrosine kinase inhibitor (TKI) approved for first-line treatment of ROS1 fusion-positive NSCLC (12, 13). According to the PROFILE1001 study, the objective response rate (ORR) of crizotinib for ROS1-positive NSCLC was 72%, with the median progression free survival (PFS) and median overall survival (OS) were 19.3 months and 51.4 months, respectively. As of now, the FDA has approved three drugs demonstrating significant efficacy against ROS1 rearranged NSCLC: crizotinib, entrectinib and repotrectinib (14–17).

High expression of PD-L1 has been reported to be associated with ROS1 rearrangement, but negatively correlated with epidermal growth factor receptor (EGFR) mutations (18, 19). Previous studies have indicated that PD-L1 expression correlates with unfavorable treatment outcomes with EGFR-TKIs (20–22). Additionally, several investigations have highlighted that positive PD-L1 expression in ALK-positive NSCLC patients undergoing crizotinib treatment is associated with adverse clinical outcomes (23–25). However, the association between PD-L1 expression and crizotinib response in ROS1-positive NSCLC remains ambiguous. Clarifying the relationship between PD-L1 expression and crizotinib treatment could aid in precisely identifying which ROS1-positive patients would derive the greatest benefit from crizotinib therapy.

In this study, we enrolled advanced NSCLC patients harboring ROS1-positive mutations who underwent crizotinib treatment as first-line therapy. Our aim was to investigate the association between PD-L1 expression and clinical characteristics and to elucidate the impact of PD-L1 expression on the clinical outcomes of first-line crizotinib therapy.

## 2 Materials and methods

### 2.1 Patients and samples

In this retrospective study, patients with ROS1-rearranged NSCLC who were treated with crizotinib as first-line therapy at the First Affiliated Hospital of Zhengzhou University between January 2016 and December 2021, were included. The inclusion and exclusion criteria for this study were as follows: Inclusion criteria:1) Patients with pathologically confirmed advanced or metastatic NSCLC; 2) ROS1 gene fusion-positive; 3) Detection of PD-L1 expression at the time of diagnosis from primary tumor or from a metastatic lesion; 4) Crizotinib administered as the first-line treatment. Exclusion criteria:1) Patients with double or multiple primary neoplasms; 2) Presence of EGFR mutations or other sensitizing mutations.

### 2.2 Study design

The data of this study were collected from clinical records on medical history or phone contact. We collected baseline characteristics of patients through medical records, including age, sex, smoking status, pathological type, disease stage, metastatic site, ROS1 fusion partner, PD-L1 expression etc. The TNM staging adhered to the guidelines set forth by the International Association for the Study of Lung Cancer (8th edition). Owing to the retrospective nature of the study, obtaining written informed consent from participants was deemed unnecessary. Patients receiving crizotinib therapy were subject to chest computed tomography evaluations at minimum intervals of three months. The assessment of therapeutic response was conducted in accordance with the Response Evaluation Criteria in Solid Tumors (RECIST) version 1.1. Individuals who had not experienced progression or death by the cut-off date of February 20, 2024, were categorized as censored at their most recent follow-up. PFS was defined as the duration from the initiation of crizotinib therapy to the inaugural documentation of tumor progression or mortality. OS was characterized as the interval from the onset of crizotinib treatment to the occurrence of death. ORR was calculated as the fraction of patients exhibiting either a complete or partial response to crizotinib, based on RECIST 1.1 criteria. The disease control rate (DCR) was defined as the percentage of patients achieving a complete response (CR), partial response (PR), or maintaining a stable disease (SD). The primary objectives of this study were to assess ORR and PFS, with a secondary objective to assess OS and DCR.

### 2.3 Detection of PD-L1 and ROS1 fusion

Tumor PD-L1 expression was assessed through immunohistochemical (IHC) staining using the PD-L1 Clone 22C3 pharmDx Kit and the Automated Link 48 platform (Dako,Carpinteria, CA). The Tumor Proportion Score (TPS) quantifies PD-L1 expression, measuring the percentage of cells exhibiting positive membrane staining among 100 cancer cells. PD-L1 expression was detected in tumor samples prior to crizotinib treatment. Patients were stratified into three categories based on their TPS scores: a PD-L1 negative group (PD-L1 < 1%), a PD-L1 low expression group (1% ≤ PD-L1 < 50%), and a PD-L1 high expression group (PD-L1 ≥ 50%). The PD-L1 positive cohort was defined by a TPS ≥ 1%, whereas the PD-L1 negative group was characterized by a TPS < 1%. Detection of ROS1 rearrangements was performed using IHC, separation fluorescence *in situ* hybridization (FISH), reverse transcription-polymerase chain reaction (RT-PCR), or next-generation sequencing (NGS).

### 2.4 Statistical analysis

Statistical analyses were conducted using GraphPad Prism version 8.0.1 (GraphPad Software, Inc.) and IBM SPSS Statistics software (IBM Corporation). The Chi-square test or Fisher’s exact test was employed to compare clinical characteristics and response rates between PD-L1 cohorts. Kaplan-Meier survival curves were constructed to illustrate the impact of PD-L1 expression on PFS and OS. The log-rank test was utilized to analyze differences between the survival curves. HRs and 95% CIs were calculated using Cox regression analysis. A P-value of <0.05 was considered to indicate statistical significance.

## 3 Results

### 3.1 Patient demographics and clinical characteristics

**Figure 1:**
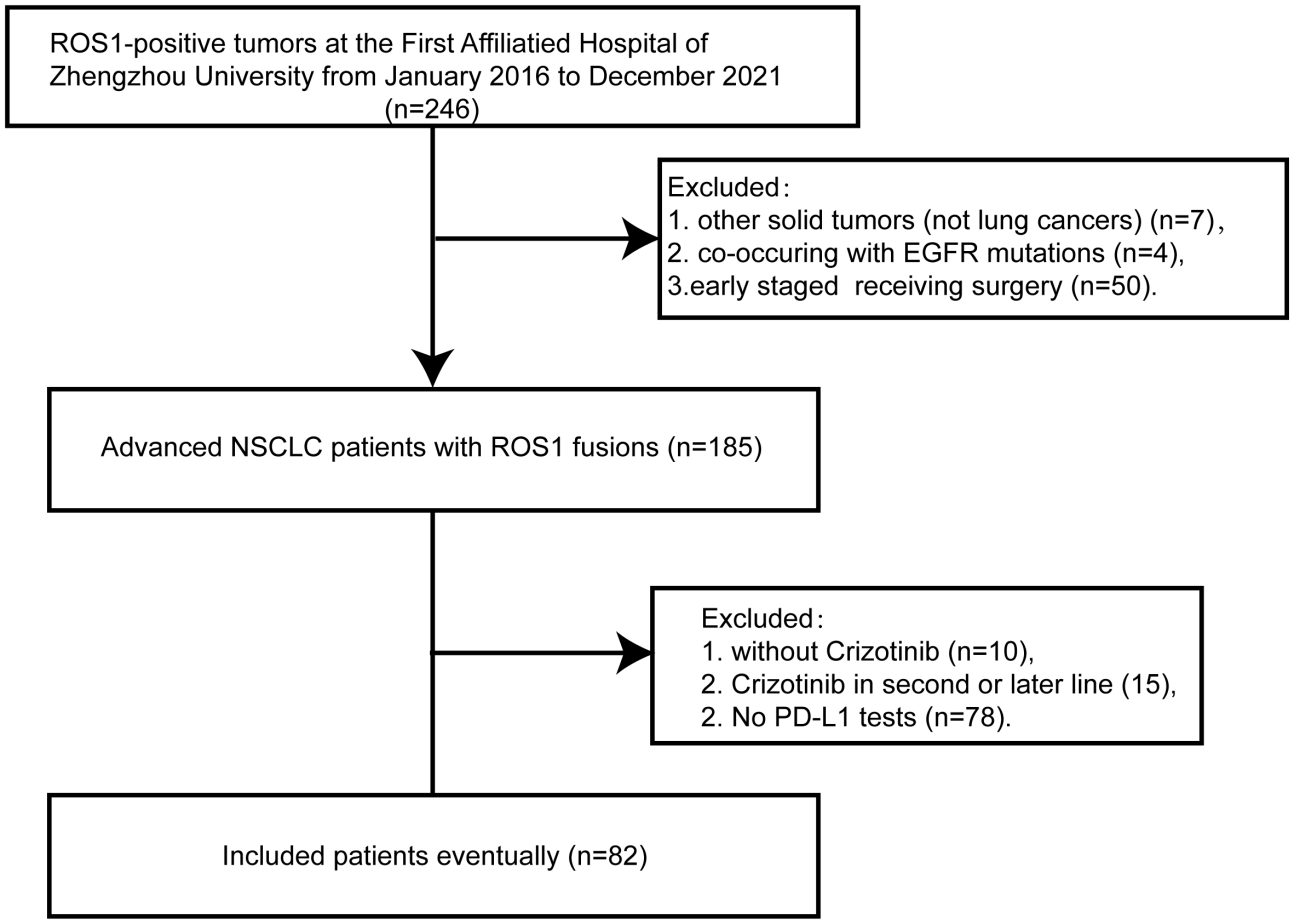
Flow chart of this study. ROS1, ROS proto-oncogene 1; EGFR, epidermal growth factor receptor;NSCLC, non-small-cell lung cancer; PD-L1, programmed death-ligand 1.

**Figure 2:**
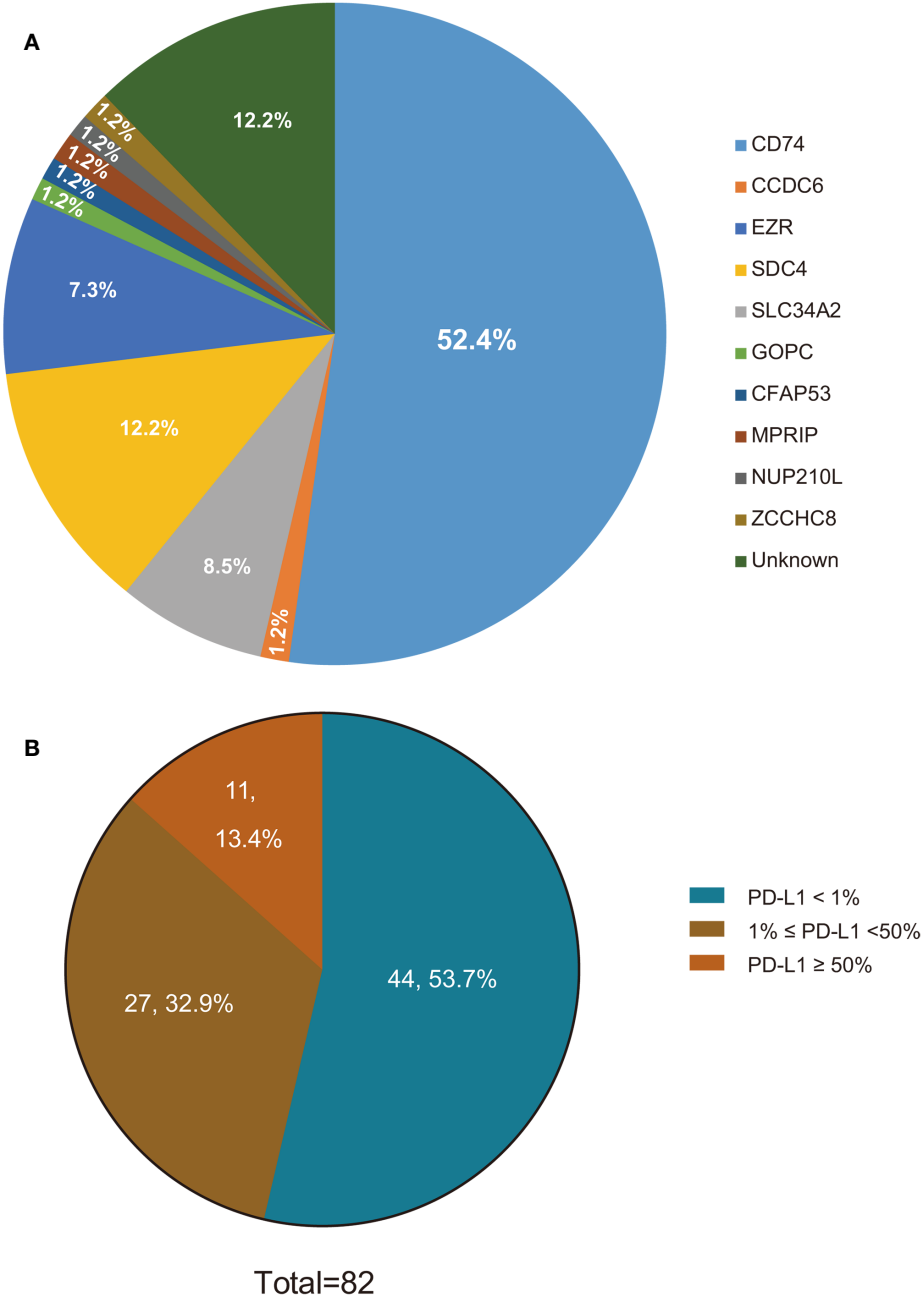
ROS1 fusion variants and PD-L1 status in ROS1-positive NSCLC patients. **(A)** ROS1 fusion variants in ROS1-positive NSCLC patients; **(B)** PD-L1 status in ROS1-positive NSCLC patients. ROS1, ROS proto-oncogene 1; PD-L1, programmed death-ligand 1; NSCLC, non-small-cell lung cancer.

Between January 2016 to December 2021, a total of 246 Chinese patients with ROS1-positive tumors were identified. Of these, 82 patients who met the inclusion criteria were ultimately enrolled in the study (**Figure 1**). All included patients were ROS1 fusion NSCLC, with 77 presenting with adenocarcinoma, 3 with squamous cell carcinoma, and 2 with adenosquamous carcinomas. The majority of the patients (87.8%) were classified as stage IV, and 10 patients were in stages IIIB or IIIC. The cohort comprised 52 females and 30 males, with a median age at diagnosis of 54 years (range: 30–78 years). A significant portion of the patients (76.8%) were never-smokers. ROS1 rearrangements were identified in 72 of the 82 patients (87.8%) through NGS, while 10 patients (12.2%) were detected by RT-PCR. Among the 72 cases with detectable ROS1 fusion variants, 10 different fusion partners were identified. As depicted in **Figure 2A**, the most prevalent ROS1 fusion partner was CD74, found in 43 of 82 samples (52.4%); other common partner genes included SDC4 (in 10 tumors), SLC34A2 (in 7 tumors), and EZR (in 6 tumors). Six rare fusion partners were also identified: GOPC, MPRIP, NUP210L, ZCCHC8, CCDC6, and CFAP53, each present in one tumor. Notably, NUP210L and CFAP53 have not been previously reported. The clinicopathological characteristics of the patients are summarized in **Table 1**.

**Table 1:** Baseline Characteristics.

Characteristics	Patients (n=82) (%)
**Age median(range), years**	54 (30–78)
Sex	
**Male**	30 (36.6)
**Female**	52 (63.4)
Smoking status	
**Never smoker**	63 (76.8)
**Former or current smoker**	19 (23.2)
Histology	
**Adenocarcinoma**	77 (93.9)
**Squamous cell carcinoma**	3 (3.7)
**Adeno-squamous carcinoma**	2 (2.4)
Stage	
**IIIB/IIIC**	10 (12.2)
**IV**	72 (87.8)
Brain metastases	
**Yes**	16 (19.5)
**No**	66 (80.5)
PD-L1 expression	
**TPS<1%**	44 (53.7)
**1%≤TPS<50%**	27 (32.9)
**TPS ≥50%**	11 (13.4)

### 3.2 Correlations between PD-L1 expression and baseline characteristics

We investigated the association between PD-L1 expression and various clinicopathological parameters, including gender, age, smoking status, TNM stage, and ROS1 fusion subtypes. As depicted in **Figure 2B**, PD-L1 expression was positive in 38 patients, with 11 patients exhibiting high PD-L1 expression, 27 patients displaying low PD-L1 expression, and the remaining 44 patients showing negative PD-L1 expression. Our study showed that PD-L1 expression was not affected by age, sex, histopathological type, tumor stage, or ROS1 fusion subtypes (**Supplementary Table 1**).

### 3.3 Association between PD-L1 expression and treatment response

**Figure 3:**
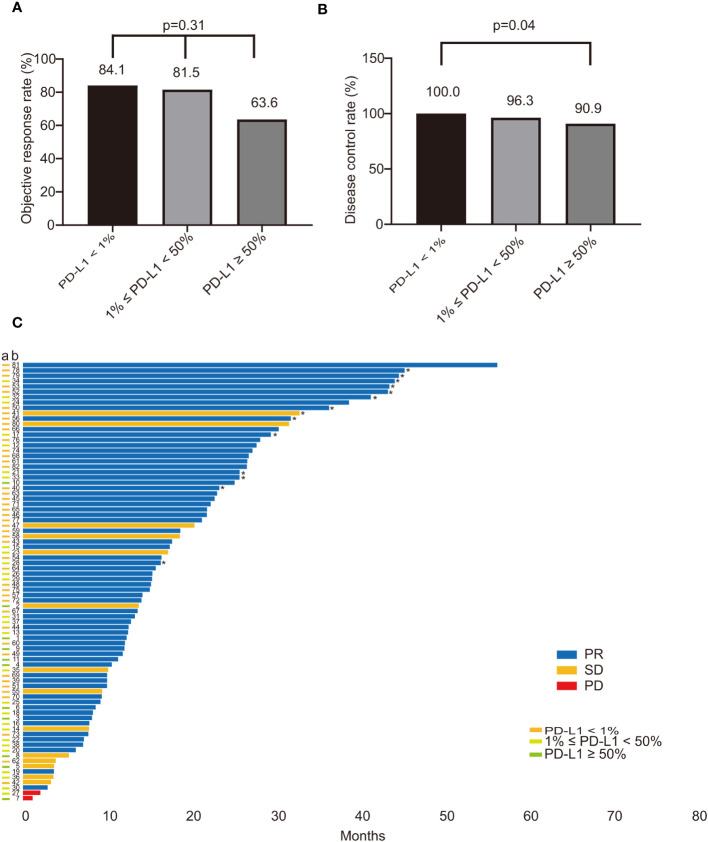
The impact of PD-L1 on the treatment response of ROS1-positive NSCLC patients treated with crizotinib. **(A)** The ORR in patients with different PD-L1 status (PD-L1 < 1% vs. 1% ≤ PD-L1 < 50% vs. PD-L1 ≥ 50%) was 84.1%, 81.5%, and 63.6%, respectively (p=0.31); **(B)** The DCR of patients compared with PD-L1 status (PD-L1 < 1% vs. 1% ≤ PD-L1 < 50% vs. PD-L1 ≥ 50%); There was a statistical difference in DCR between the PD-L1 negative group and the PD-L1 high expression group, which were 100% and 90.9%, respectively (p=0.04); **(C)** The survival plot illustrating the treatment response and PFS of each patient. The bar colors of blue, yellow, and red refer to PR, SD, PD. The different colors of the “a” column on the vertical axis represent the PD-L1 expression status. "*" indicates that the patient was not resistant to crizotinib treatment at the last follow-up. PD-L1, programmed death-ligand 1; ROS1, ROS proto-oncogene 1; NSCLC, non-small-cell lung cancer; ORR, objective response rate; DCR, disease control rate; PFS, progression free survival; PR, partial response; SD, stable disease; PD, progressive disease.

The ORR for all included patients was 80.5%. The ORR of the PD-L1 negative group was 84.1%, while the PD-L1 positive group had an ORR of 76.3% (p=0.38), as depicted in **Supplementary Figure 1A**. When patients were stratified into three groups based on TPS values, the ORR of the PD-L1 negative group and the PD-L1 low expression group were found to be similar, at 84.1% and 81.5%, respectively. However, the ORR of the PD-L1 high expression group was lower, at only 63.6% (p=0.31), as illustrated in **Figure 3A**. Consequently, no statistically significant difference in ORR was observed among ROS1 rearrangement NSCLC patients with varying PD-L1 expression statuses. The DCR of the PD-L1 positive and PD-L1 negative groups were 94.7% and 100%, respectively, as shown in **Supplementary Figure 1B**. Conversely, a significant difference in DCR was noted between the PD-L1 negative group and the PD-L1 high expression group, which were 100% and 90.9%, respectively (p=0.04), as presented in **Figure 3B**. **Figure 3C** illustrates the treatment response and PFS of each patient. Moreover, PD-L1 expression did not differ between responders (best response of PR) and non-responders (best response of SD and progressive disease (PD)), as demonstrated in **Supplementary Figure 1C**.

### 3.4 Association between PD-L1 expression and clinical outcome

**Figure 4:**
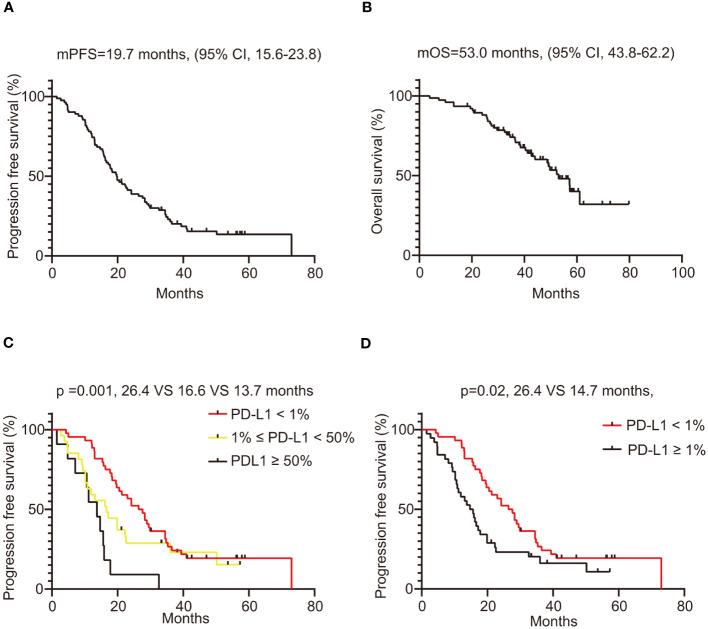
Survival analysis of ROS1-positive NSCLC patients treated with crizotinib. **(A)** PFS of all included patients, with 95% confidence intervals indicated; **(B)** OS of all included patients, with 95% confidence intervals indicated; **(C)** The PFS of ROS1-positive lung cancer patients compared by PD-L1 status (PD-L1 < 1% vs. 1% ≤ PD-L1 < 50% vs. PD-L1 ≥ 50%); **(D)** The PFS of ROS1-positive lung cancer patients compared by PD-L1 status (negative vs. positive). ROS1, ROS proto-oncogene 1; NSCLC, non-small-cell lung cancer; PFS, progression free survival; OS, overall survival; PD-L1, programmed death-ligand 1.

The median PFS of patients treated with crizotinib was 19.7 months (95% confidence interval [CI], 15.6 - 23.8). The survival plots depicting PFS related to crizotinib treatment are illustrated in **Figure 4A**. Kaplan-Meier analysis revealed that the median PFS of patients in the PD-L1 negative group, PD-L1 low expression group, and PD-L1 high expression group were 26.4, 16.6, and 13.7 months, respectively (p=0.001) (**Figure 4C**). The median PFS in the PD-L1-negative group was 26.4 months, compared to 14.7 months in the PD-L1-positive group, with a statistically significant difference between these two groups (p=0.02) (**Figure 4D**). In univariate analysis, brain metastases (p=0.06) and non-adenocarcinoma pathologic types (p=0.09) tended to be associated with shorter PFS, but did not achieve a statistical difference, as shown in **Table 2**. In the multivariate Cox regression analysis model, which included all factors with p<0.1 in the univariate analysis, PD-L1 expression status (p=0.02) and brain metastasis (p=0.04) showed a significant association with PFS (**Table 2**). Overall, our findings indicate that in ROS1-positive NSCLC patients treated with crizotinib, those with positive PD-L1 expression experienced shorter PFS compared to patients with negative PD-L1 expression.

**Table 2:** Univariate analysis and multivariate analysis of PFS of ROS1-positive NSCLC patients receiving crizotinib.

Characteristics	Univariate analysis		Multivariate analysis	
	mPFS(m)	P value	HR (95% CI)	P value
Age<65 vs. Age≥65	19.5 vs 20.5	0.94		
Female vs. male	19.7 vs 18.2	0.61		
Never smoker vs. Former or current smoker	19.9 vs 17.7	0.47		
PD-L1 negative vs. positive	26.4 vs 14.7	0.02	0.575(0.36–0.93)	0.02
IIIB/IIIC vs. IV stage	15.7 vs 20.5	0.16		
Adenocarcinoma vs. Non-adenocarcinoma	19.9 vs 13.1	0.09	0.41(0.16–1.05)	0.06
Brain metastasis no vs. yes	19.9 vs 15.6	0.06	0.549(0.31–0.96)	0.04
Bone metastasis no vs. yes	22.6 vs 17.8	0.15		

We further analyzed the association between PD-L1 expression and OS in ROS1-positive patients treated with crizotinib. The survival plots depicting OS related to crizotinib treatment are illustrated in **Figure 4B**. The median OS of the entire population was 53.0 months (95% CI, 43.8 - 62.2). The median OS of the PD-L1 negative group was longer than that of the PD-L1 positive group, at 57.2 months and 53.0 months, respectively, but the difference was not statistically significant (p=0.43), as presented in **Supplementary Figure 2B**. Furthermore, when patients were stratified into PD-L1 negative, PD-L1 low expression, and PD-L1 high expression groups, there was still no statistically significant difference in OS among the three groups (p = 0.71) (**Supplementary Figure 2A**). Overall, patients with positive PD-L1 expression tended to have shorter OS compared to those with negative PD-L1 expression, but this difference was not statistically significant.

## 4 Discussion

The management of advanced lung cancer has evolved into the era of precision medicine (26, 27). The selection of an appropriate treatment regimen is determined not merely by the histological subtype but also by the presence of specific driver mutations and the level of PD-L1 expression (28, 29). Consequently, the precise detection of biomarkers is essential for the customization of therapy on an individual basis.

Accumulating evidence suggests that the regulation of PD-L1 expression is intricately linked to oncogenic mutations driving NSCLC (30). Evans et al. (2018) conducted PD-L1 detection with the 22C3 Assay on 10005 NSCLC patients, and the results showed that poorly-differentiated tumor histology and ALK translocation were significantly associated with PD-L1 expression (31). Another study examined PD-L1 in 130 consecutive NSCLC samples and found that PD-L1 expression (TPS≥1%) was significantly associated with wild-type EGFR, and ROS1 rearrangement was associated with high PD-L1 expression (19). The above studies suggest that mutations in the EGFR gene are associated with diminished PD-L1 expression, whereas higher incidences of PD-L1 positivity are observed in NSCLC patients harboring ALK or ROS1 rearrangements (31–33). The latter, through the activation of receptor tyrosine kinases, significantly enhances NSCLC cell proliferation (8). Furthermore, the escalation of PD-L1 expression in NSCLC has been correlated with the activation of the RAS signaling pathway, a process in which the ROS1 receptor tyrosine kinase plays a pivotal role (34). Our research findings indicate that 46.3% of NSCLC patients with ROS1 rearrangements exhibit PD-L1 positivity, with 13.4% displaying high levels of PD-L1 expression and 32.9% showing lower levels. These observations underscore the association between ROS1 rearrangement and elevated PD-L1 expression in NSCLC, aligning with existing literature that points to the abnormal activation of oncogenes, including ROS1 and ALK, as a driving force behind PD-L1 expression in this disease context (18, 35).

With the elucidation of the complex interaction between the immune system and the activation of oncogenic driver genes, the role of PD-L1 expression as a predictive biomarker for targeted therapies has received increasing attention. Accumulating evidence has identified PD-L1 expression as an adverse prognostic indicator in patients harboring EGFR mutations, irrespective of the application of targeted drug therapies (20, 21). Historically, the influence of PD-L1 on the effectiveness of targeted treatments in EGFR-mutant NSCLC has been a focal point of research. Notably, recent comprehensive analyses reveal that elevated PD-L1 levels correlate with diminished efficacy of EGFR-TKIs in EGFR-mutated NSCLC (36). A study on patients with ALK-positive NSCLC treated with crizotinib reported that the absence of PD-L1 expression is linked to prolonged PFS (24). However, divergent findings from another investigation suggest that initial PD-L1 levels do not significantly forecast the success of alectinib therapy in individuals with ALK-positive lung cancer (25).

To our knowledge, this constitutes the inaugural investigation into the correlation between PD-L1 expression and crizotinib’s therapeutic efficacy. Our study demonstrates a significant association between PD-L1 expression and reduced PFS in patients with advanced NSCLC harboring ROS1 fusions. Conversely, although patients with positive PD-L1 expression exhibited a trend towards decreased OS compared to those with negative PD-L1 expression, this observation did not reach statistical significance. These findings propose that PD-L1 may serve more effectively as a predictive biomarker for crizotinib-induced PFS, rather than as a prognostic biomarker for OS. Investigating the immune mechanisms underlying TKI resistance and exploring potential synergies with combination immunotherapy could enhance crizotinib’s therapeutic impact on NSCLC patients with ROS1 fusion.

The underlying mechanism of immune microenvironment-mediated TKI resistance in oncogene-driven lung cancer is still under investigation. Recent research indicates that in melanoma driven by B-type Raf kinase (BRAF) mutations, resistance to inhibitors of the mitogen-activated protein kinases (MAPK) pathway may arise through macrophage-secreted Tumor Necrosis Factor alpha (TNF-α) (37). Concurrently, elevated expression of PD-L1 has been observed in cell lines resistant to BRAF inhibitors as well as in samples from patients, hinting at a potential role of PD-L1 in conferring resistance to targeted therapies (38–40). Furthermore, lung cancer specimens and cell lines that developed resistance to ALK inhibitors exhibited increased PD-L1 expression, linking the PD-1/PD-L1 pathway to ALK inhibitor resistance in cases of ALK rearrangement (41). Liu et al. (2020) demonstrated that in NSCLC resistant to crizotinib, ROS1 rearrangements lead to an upregulation of PD-L1 expression via the mitogen-activated protein kinase kinase (MEK) - extracellular signal-regulated kinases (ERK) signaling pathway (18). This evidence supports the hypothesis that blocking the PD-L1/PD-1 pathway could represent a promising approach for treating crizotinib-resistant NSCLC with ROS1 rearrangement. However, The efficacy of immunotherapy in ROS1 fusion NSCLC patients remains to be further studied. We have reported a ROS1 positive NSCLC patient, who achieved a continuous PR to immunotherapy plus chemotherapy and a more than 35 months PFS (42). Nevertheless, another study indicated that ROS1 fusion NSCLC patients treated with immunotherapy alone displayed an unsatisfactory ORR of 16.7% (43). Chemotherapy combined with immunotherapy with or without bevacizumab may be one of the treatment options for ROS1 positive NSCLC patients after TKI failure (44). A large number of studies have shown that NSCLC patients with high PD-L1 expression are more likely to benefit from immunotherapy (45). Immunotherapy combined with chemotherapy after TKI resistance may be a reason why PD-L1 expression was not associated with the OS of the crizotinib treatment in our study.

Several limitations warrant mention within the context of this study. Initially, the retrospective nature of this study inherently introduces selection bias. Additionally, the relatively rare occurrence of ROS1 fusion in NSCLC constrained the size of the patient cohort, potentially limiting the representativeness of our sample for the broader ROS1 fusion-positive population. Moreover, patients treated with other TKIs, such as entrectinib or ceritinib, were excluded from our analysis, leaving the relationship between PD-L1 expression and the prognosis with these other TKIs an open question for future research. Consequently, expansive studies with larger cohorts are essential to substantiate our findings.

In summary, our investigation reveals that among ROS1-positive NSCLC patients treated with crizotinib as an initial therapeutic approach, PD-L1 expression may serve as an adverse prognostic marker for PFS rather than OS. Consequently, assessing PD-L1 expression in patients with ROS1-positive NSCLC could offer valuable insights for predicting clinical outcomes.

## Data availability statement

The original contributions presented in the study are included in the article/**Supplementary Material**. Further inquiries can be directed to the corresponding authors.

## Ethics statement

The studies involving humans were approved by Ethics Committee of the First Affiliated Hospital of Zhengzhou University (2024-KY-0231-002). The studies were conducted in accordance with the local legislation and institutional requirements. The ethics committee/institutional review board waived the requirement of written informed consent for participation from the participants or the participants’ legal guardians/next of kin because our study was a retrospective study.

## Author contributions

HZ: Conceptualization, Data curation, Formal analysis, Funding acquisition, Investigation, Methodology, Project administration, Resources, Software, Supervision, Validation, Visualization, Writing – original draft, Writing – review & editing. ZZ: Conceptualization, Data curation, Investigation, Methodology, Supervision, Writing – original draft, Writing – review & editing. NY: Conceptualization, Formal analysis, Investigation, Methodology, Project administration, Software, Supervision, Validation, Writing – original draft, Writing – review & editing. XL: Conceptualization, Funding acquisition, Investigation, Methodology, Resources, Visualization, Writing – original draft, Writing – review & editing.
