# Supplementary material for: Association of PD-L1 expression and clinical outcomes in ROS1 - rearranged advanced non-small cell lung cancer treated with crizotinib
Source: Front Oncol. 2024 May 21;14:1405683. doi: 10.3389/fonc.2024.1405683 (PMC11148223; doi:10.3389/fonc.2024.1405683)
Supplement: Supplementary file 1 [file DataSheet_1.doc]

**Supplementary Materials**

**Supplemental Table 1.** The association between baseline characteristics and PD-L1 expression status.

| Characteristics | PD-L1 Negative  n (%) | PD-L1 Positive  n (%) | P value |
| --- | --- | --- | --- |
| Age (years) |  |  |  |
| ≥65 | 9 (20.5) | 4 (10.5) | 0.2020 |
| <65 | 35 (79.5) | 34 (89.5) |  |
| Sex |  |  |  |
| Male | 18 (40.9) | 12 (31.6) | 0.3817 |
| Female | 26 (59.1) | 26 (68.4) |  |
| Smoking status |  |  |  |
| Never smoker | 34 (77.3) | 29 (76.3) | 0.9184 |
| Former or current smokers | 10 (22.7) | 9 (23.7) |  |
| Histology |  |  |  |
| Adenocarcinoma | 42 (95.5) | 35 (92.1) | 0.5274 |
| others | 2 (4.5) | 3 (7.9) |  |
| Stage |  |  |  |
| IIIB/IIIC | 4 (9.1) | 6 (15.8) | 0.3553 |
| IV | 40 (90.9) | 32 (84.2) |  |
| Brain metastases |  |  |  |
| Yes | 9 (20.5) | 7 (18.4) | 0.8168 |
| No | 35 (79.5) | 31 (81.6) |  |
| ROS1 fusion subtypes |  |  |  |
| CD74 | 27 (67.5) | 16 (50) | 0.1325 |
| Others | 13 (32.5) | 16 (50) |  |

**Supplemental Figure 1**

**
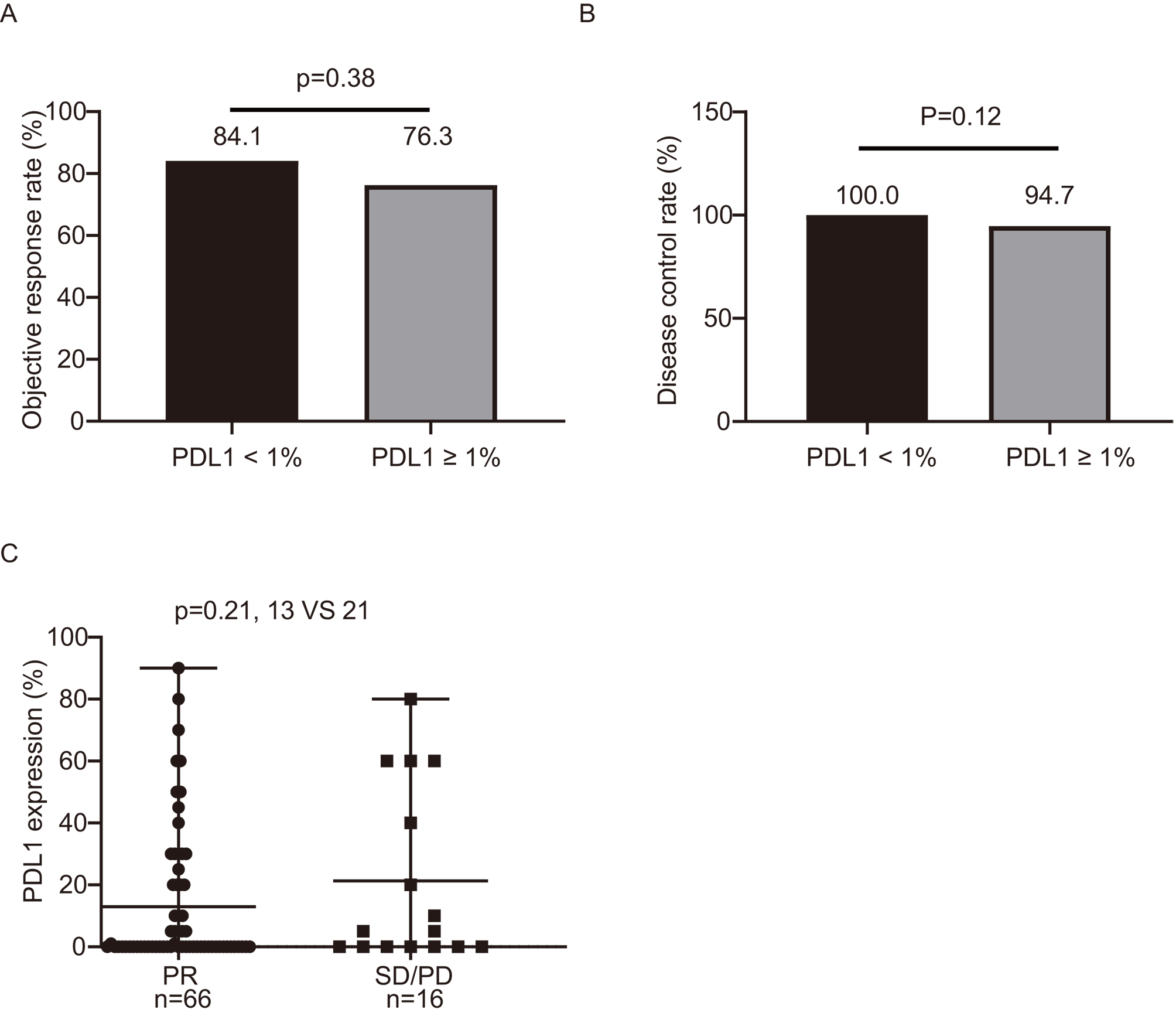
**

**Supplemental Figure 1** The impact of PD-L1 on the treatment response of ROS1-positive NSCLC patients treated with crizotinib. (A) The ORR of ROS1-positive lung cancer patients compared by PD-L1 status (negative vs. positive); (B) The DCR of ROS1-positive lung cancer patients compared by PD-L1 status (negative vs. positive); (C) The relationship between PD-L1 expression and efficacy in responders (best response PR) and non-responders (best response SD and PD). Abbreviations: PD-L1, programmed death-ligand 1; ROS1, ROS proto-oncogene 1; NSCLC, non-small-cell lung cancer; ORR, objective response rate; DCR, disease control rate; PR, partial response; SD, stable disease; PD, progressive disease.

**Supplemental Figure 2**

**
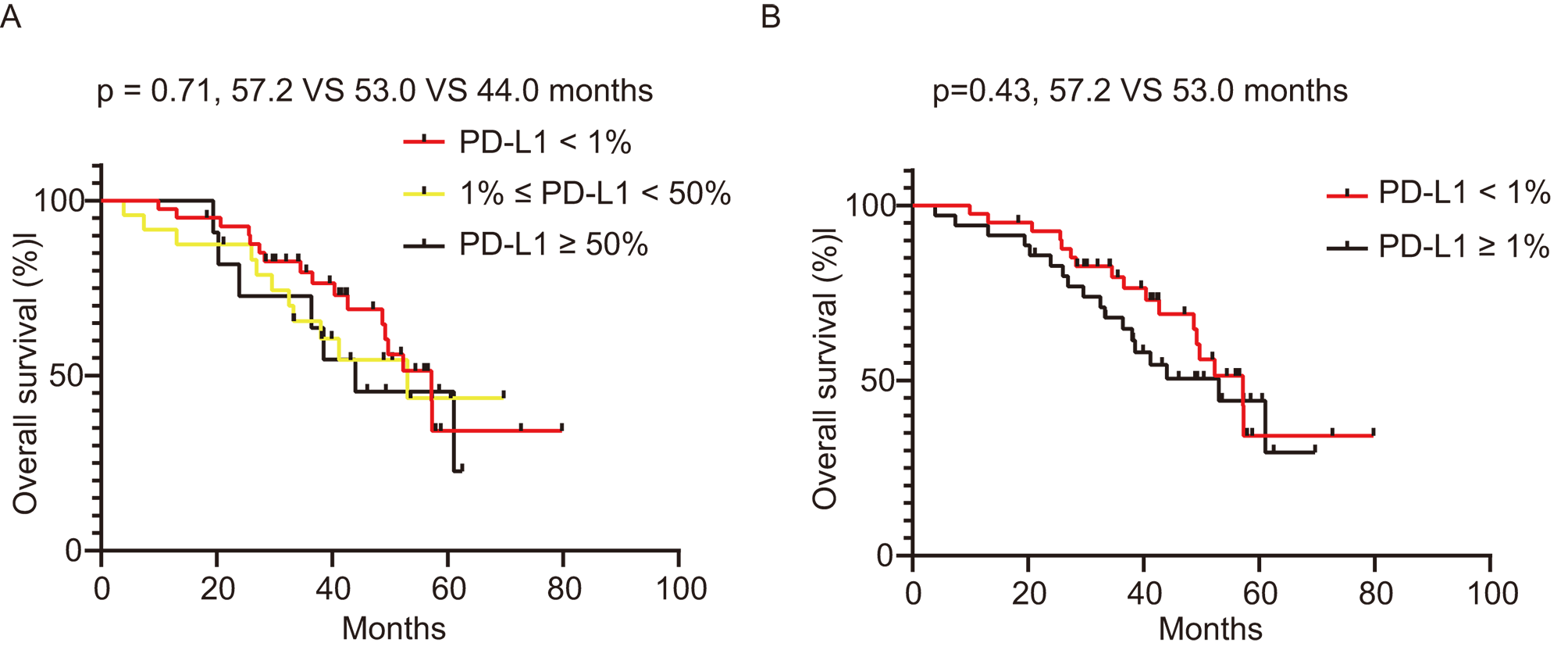
**

**Supplemental Figure 2.** The impact of PD-L1 on the OS of ROS1-positive NSCLC patients treated with crizotinib. (A) The OS of ROS1-positive lung cancer patients compared by PD-L1 status (PD-L1 < 1% vs. 1% ≤ PD-L1 < 50% vs. PD-L1 ≥ 50%); (D) The OS of ROS1-positive lung cancer patients compared by PD-L1 status (negative vs. positive). Abbreviations: PD-L1, programmed death-ligand 1; OS, overall survival; ROS1, ROS proto-oncogene 1; NSCLC, non-small-cell lung cancer.
